# Downregulation of caspase-3 alleviates *Mycoplasma pneumoniae*-induced apoptosis in alveolar epithelial cells

**DOI:** 10.3892/mmr.2021.12456

**Published:** 2021-09-22

**Authors:** Shan Shi, Xiaolei Liu, Haibo Li

Mol Med Rep 16: 9601-9606, 2017; DOI: 10.3892/mmr.2017.7782

Following the publication of the above paper, the authors contacted the Editorial Office to request that the article be retracted on account of the presented results being unreliable; essentially, the authors did not repeat their experiments the requisite number of times, and they were unable to identify in subsequent experiments any increase in the apoptotic rate of the alveolar epithelial cells as had been reported. The Editor of *Molecular Medicine Reports* has agreed that this paper should be retracted from the Journal. The Editor and the authors apologize to the readership for any inconvenience caused.

